# Molecular mechanisms of heterogeneous oligomerization of huntingtin proteins

**DOI:** 10.1038/s41598-019-44151-0

**Published:** 2019-05-20

**Authors:** Silvia Bonfanti, Maria Chiara Lionetti, Maria Rita Fumagalli, Venkat R. Chirasani, Guido Tiana, Nikolay V. Dokholyan, Stefano Zapperi, Caterina A. M. La Porta

**Affiliations:** 1 Center for Complexity and Biosystems, Department of Physics, University of Milan, Via Celoria 16, 20133 Milano, Italy; 2 Center for Complexity and Biosystems, Department of Environmental Science and Policy, University of Milano, via Celoria 26, 20133 Milano, Italy; 3 Departments of Pharmacology and Biochemistry & Molecular Biology, Penn State College of Medicine, Hershey, PA 17033 USA; 4 CNR - Consiglio Nazionale delle Ricerche, Istituto di Chimica della Materia Condensata e di Tecnologie per l’Energia, Via R. Cozzi 53, 20125 Milano, Italy

**Keywords:** Huntington's disease, Computational biophysics, Biological physics

## Abstract

There is still no successful strategy to treat Huntington’s disease, an inherited autosomal disorder associated with the aggregation of mutated forms of the huntingtin protein containing polyglutamine tracts with more than 36 repeats. Recent experimental evidence is challenging the conventional view of the disease by revealing transcellular transfer of mutated huntingtin proteins which are able to seed oligomers involving wild type forms of the protein. Here we decipher the molecular mechanism of this unconventional heterogeneous oligomerization by performing discrete molecular dynamics simulations. We identify the most probable oligomer conformations and the molecular regions that can be targeted to destabilize them. Our computational findings are complemented experimentally by fluorescence-lifetime imaging microscopy/fluorescence resonance energy transfer (FLIM-FRET) of cells co-transfected with huntingtin proteins containing short and large polyglutamine tracts. Our work clarifies the structural features responsible for heterogeneous huntingtin aggregation with possible implications to contrast the prion-like spreading of Huntington’s disease.

## Introduction

Conformational diseases, such as Alzheimer’s, Parkinson’s and Huntington’s diseases, are part of an increasingly common class of neurological disorders characterized by the aggregation of aberrant conformations of proteins. In particular, Huntington’s disease (HD) has autosomal dominant inheritance and is caused by mutations leading to an abnormal expansion in the polyglutamine (polyQ) tract of the huntingtin (HTT) protein, leading to the formation of HTT inclusion bodies in the brain^1,2^. The severity of the illness may depends on the polyQ repeat length and the disease arises only in subjects where the polyQ region displays 36 repeats or more^3–5^. The molecular mechanism of Huntington’s disease has not been completely elucidated, but the general consensus is that the polyQ expansion in the HTT gene’s first exon is susceptible to atypical folding behaviors that somehow interfere with normal neuronal functions or survival^6^. Expressing only the first exon of the HTT gene is enough to observe HD pathogenesis in mice and toxicity in cells^7^.

Although Huntington’s disease is a cell autonomous genetic disorder, recent experimental work, both *in vitro* and *in vivo*, shows that mutant huntingtin proteins can be transmitted to neighboring cells^8–11^ with potential prion-like infection and propagation^12,13^. Remarkably, transcellular transmission of mutated huntingtin proteins leads to heterogeneous aggregation with wild type forms of the protein^9,10^. Considerable experimental^4,14,15^ and computational^5,16,17^ efforts have been devoted to elucidate the molecular mechanism underlying homogeneous aggregation of HTT in a polyQ dependent manner, while heterogeneous aggregation of HTT proteins with different lengths of the polyQ tract is much less studied^18,19^, despite its fundamental relevance for a possible prion-like disease expansion.

Much of the knowledge on polyQ-dependent aggregation has been derived from model peptides with numerous studies conducted on isolated polyQ monomers and oligomers^4^. The main problem is that the structural properties of monomeric polyQ are still unclear and inconsistent^20^. Experiments on single polyQ tracts of different lengths, modified for solubility, suggest high conformational flexibility (a random coil, a collapsed structure, an *α*-helix, a *β*-sheet, or even a PP II helix) and a dependence of its structure on the local environment. It is generally believed that polyQ aggregates adopt structures rich in *β*-sheet content with longer polyQ peptides aggregating faster.

The aggregation process in the cell is much more complex than for isolated polyQ tracts and depends also on the environment. To mimic the environment surrounding the polyQ regions in full-length proteins, some authors fused other soluble proteins to polyQ^21^. It is important to understand the relative contributions of the polyQ stretch and of the rest of the structure in modulating aggregation. Molecular dynamics simulations found a positive correlation between the length of the polyQ expansion and its probability to form a *β*-rich misfolded state^16^. On the basis of these simulations, it was concluded that the flanking sequences affect the formation of *β*-sheet structures in the polyQ region^16^. More recent simulations were able to reconstruct the free-energy landscape of the polyQ-dependent homogeneous HTT exon1 oligomerization^5,17^, investigating modifications of the structure by addition of specific residuals that enhance or inhibit oligomerization^5^. Molecular regions that inhibit heterogeneous HTT aggregation are however unknown, due to the lack of simulations for this case.

Here we investigate heterogeneous oligomerization of wild type HTT proteins with short polyQ tracts (*Q* < 36) and mutated ones with long polyQ tracts (*Q* > 36) numerically by discrete molecular dynamics simulations and experimentally by fluorescence-lifetime imaging microscopy/fluorescence resonance energy transfer (FLIM-FRET) in cotransfected HeLa cells. Discrete molecular dynamics simulations allow to identify the the atomic mechanisms of oligomer formation and the main determinants of their stability, providing a list of possible molecular targets that could be exploited to inhibit aggregation.

## Results

### Discrete molecular dynamics simulations reveal structural features of huntingtin oligomers

To investigate heterogeneous oligomerization between mutant and non-mutant HTT proteins, we use discrete molecular dynamics (DMD), an event-driven simulation which employs a discrete potential energy that relies on the calculation of atomic collisions (for details see the Materials and Methods section)^22–24^. This technique has already been used to efficiently study the protein folding thermodynamics and protein oligomerization and allows for a good equilibration of the structures, as shown in Fig. S1 ^25^. We focus our analysis on the HTT exon1, known to be responsible for the aggregation of mutant HTT^6^, and consider the interaction of two protein fragments with different length for the polyQ region. In particular, we consider combination of fragments of HTT-23Q, as a proxy for the wild type HTT, and HTT-74Q representing the mutant form of HTT. The Exon 1 amino acid sequence (reported in the SI) is composed by a first segment of 17 amino acids (HTT) followed by the glutamine repeat (polyQ) of length *n* = 23 or 74, a 11- (P11) and 10-proline (P10) stretch, interspersed with other regions of residues labeled as R17 and R12.

**Figure 1:**
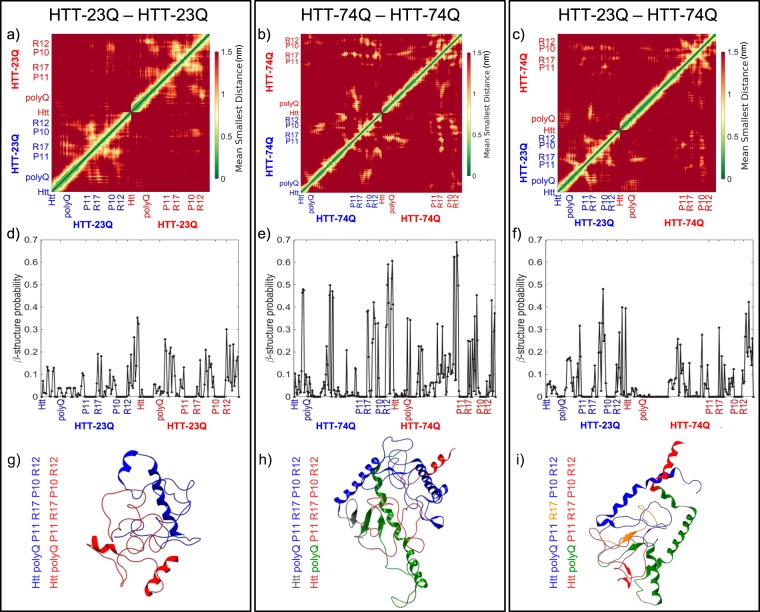
Discrete molecular dynamics simulations display *β* structure formation indicative heterogeneous HTT dimer formation. From left to right the three panels refer to HTT-23Q–HTT-23Q, HTT-74Q–HTT-74Q and HTT-23Q–HTT-74Q systems. (**a–c**) The mean smallest distance maps between different residue regions. (**d–f**) The *β* probability per-residue for the different residue regions. Error bars on each point are smaller than ${\rm{\Delta }}p=0.015$. (**g–i**) Most favorable conformational structures obtained via cluster analysis. The regions of interaction between two respective proteins having *β*-cross conformations, the precursors of aggregation, are colored differently from the rest of the protein.

Figure 1 summarizes our results on the interaction between the simulated binary systems of Human HTT Exon 1 depending on the length of the polyglutamine chain (i.e. 23Q or 74Q). Panels from left to right refer respectively to systems HTT-Q23–HTT-Q23, HTT-74Q–HTT-74Q, and the combination of the wild-type and mutant protein, HTT-Q23–HTT-74Q. All plots refer to a low simulation temperature corresponding to physiological conditions. Figure 1a–c reports the maps of the mean equilibrium distance between different residues. Secondary structures involving the two systems are observed in the off-diagonal squares, especially for the heterogeneous system, HTT-Q23–HTT-74Q. Studying these contact maps, we can infer information about regions that tend to be close and that could thus be involved in the oligomerization process. We define an aggregate precursors as a conformation displaying a *β* sheet that involves both proteins. To localize putative *β* sheet locations, we report in Fig. 1d–f the probability to find a *β* in the three systems considered.

For the HTT-Q23–HTT-Q23 binary system, the probability of *β*-sheet conformations in the polyQ region is lower in the first HTT-Q23 system than in the second one: 5% versus 20%, whereas the probability to form *β* structures in R12 and R17 domains is higher in both proteins. In the case of a pair of mutant HTT (HTT-74Q–HTT-74Q), the probability of *β*-sheet conformations is larger than in the other systems, as expected. Continuous stretches of high beta probability (≥50%) are found in both polyQ, R12 and R17 regions, with high probability also in the N terminal of the first 74Q protein. Finally, Fig. 1f displays the per-residue *β* probability for the heterogeneous system (HTT-Q23–HTT-74Q). The probability of *β*-sheet in the polyQ region of HTT-Q23 drastically increases with respect to the HTT-Q23–HTT-Q23 system, forming a stretch from residues 22 to 30. Furthermore, a high probability to find *β*-structure is found in the R12 and R17 domains of both proteins.

### Cluster analysis identifies the most probable oligomer structures

In Fig. 1g–i, we show the most likely conformation resulting from the cluster analysis^26^ (see the Materials and Methods section). The color code for the different regions of the proteins is shown through a schematic box at the bottom of the panels. The two different proteins are colored in red or blue and reflect the color notation used for the axis label of the related plots. The putative oligomerization domain (a *β* shared among two proteins) is superimposed on the protein with a different color. Fig. S2a shows the number of structures for each identified cluster for the HTT-Q23–HTT-Q23 binary system. The central structures of the first 9 clusters are reported. The most probable cluster consists of more than 100 conformations. The most favorable structure, reported in Fig. 1g displays no cross *β*-sheet (Fig. S2), suggesting a lack of oligomerization for wild type HTT. We eventually find with a lower probability that the second cluster displays interaction between R12 of the first HTT and polyQ of the second one. The same interaction is then found again with even lower probability in the sixth cluster.

Figure S2b reports the number of structures for each identified cluster for the HTT-74Q–HTT-74Q binary system. The central structures of the first 7 clusters are reported. Differently from the HTT-Q23–HTT-Q23 case, here there is a single dominant cluster with more than 1000 structures whose conformation is reported in Fig. 1h. A *β*-cross interaction is found between the Htt terminal of the first HTT-74Q and polyQ domain of the second HTT-74Q. The clustering analysis indicates also the possibility of interaction between the polyQ of the two proteins. Finally, Fig. S2c displays the number of structures for each identified cluster for the HTT-Q23–HTT-74Q binary system. The central structures of the first 5 clusters are reported. Here, there is a single dominant cluster with more than 1000 structures whose conformation is reported in Fig. 1i where *β*-cross interactions are found between the R17 domain of HTT-Q23 and polyQ of HTT-74Q.

### Simulations reveal that a single mutant protein can aggregate two wild type forms

**Figure 2:**
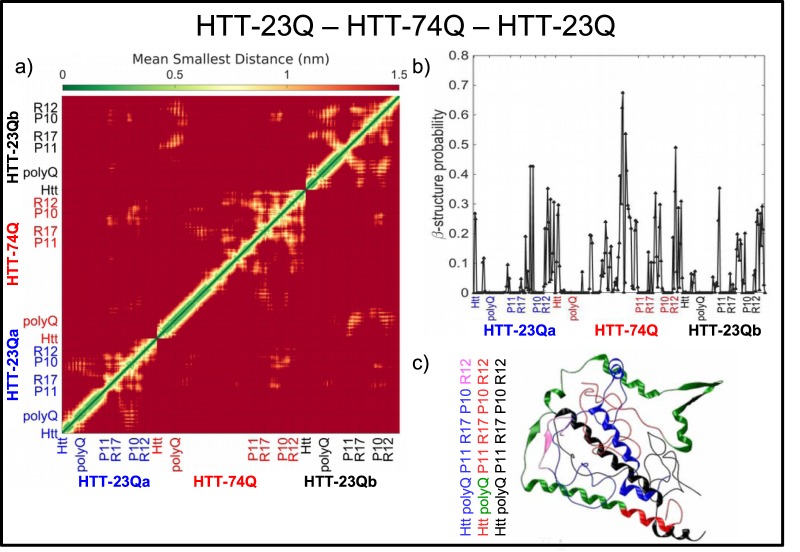
Discrete molecular dynamics simulations indicate that a single HTT-74Q protein can trigger oligomerization of two HTT-23Q proteins. (**a**) Mean smallest distance map between different residue regions for the system HTT-23Q–HTT-23Q–HTT-74Q. (**b**) Per-residue *β* probability for the different residue regions. Error bars on each point are smaller than ${\rm{\Delta }}p=0.015$. (**c**) Most favorable conformational structure obtained via cluster analysis. The regions of interaction between the respective proteins having *β*-cross conformations, the precursors of aggregation, are colored differently from the rest of the protein.

Our simulations show that the binary system HTT-Q23–HTT-74Q can form aggregate precursors. We therefore investigate if this feature can be observed for a ternary system of Human HTT Exon 1, HTT-Q23–HTT-Q23–HTT-74Q. For the sake of clarity, we distinguish the two copies of the wild type protein with the notation HTT-23Qa and HTT-23Qb. Figure 2a displays the mean distance as a function of the residue region. Again, closest cross-constructs between the possible pair combinations (HTT-23Qa–HTT-74Q, HTT-23Qa–HTT-23Qb, HTT-23Qb–HTT-74Q) can be observed in the off-diagonal squares. Figure 2b reports the probability of *β*-sheets. For the HTT-74Q this probability is highest in the polyQ region and its values are larger here than in the HTT-23Q–HTT-74Q and HTT-74Q–HTT-74Q binary systems (compare with Fig. 1). Furthermore, both HTT-23Qa and HTT-23Qb proteins show higher *β* probability in the R12 and R17 regions and in the last part of the polyQ tract (around residue 38 and especially 270). The most favorable *β*-cross interactions in the ternary oligomer occur between HTT-23Qa and HTT-74Q, and in particular region R12 in HTT-23Qa with the polyQ of HTT-74Q (conformation shown in Fig. 2c), and among the three proteins (Fig. S3). The Htt-N terminal of HTT-23Qa aggregates with R12 of HTT-23Qb, and the Htt-N terminal of HTT-23Qb aggregates with the polyQ of the 74Q protein.

### Estimates of aggregate stability identifies possible targets

To decipher the structural mechanisms for HTT oligomerization and to identify the hot spots in HTT proteins which trigger the aggregation process, we calculate the destabilization effect of point mutations, evaluating the change in free energy (ΔΔ*G*) upon mutation^27^, as discussed in the Materials and Methods section. We consider the representative structures of oligomers obtained from the cluster analysis and look for the most important interactions between monomeric subunits. Calculations are performed for all the cases considered in this work.

For the heterogeneous case, visual inspection of HTT-23Q–HTT-74Q oligomer structure (see Fig. 1i) shows *β*-cross interactions between anti-parallel *β*-sheets formed by a poly-Q region (in HTT-23Q) and the P11 region (in HTT-74Q). A smaller *β*-cross interaction is observed also between R17 of HTT-23Q and polyQ of HTT-74Q. It is worth noting here that the amount of aggregation is higher in HTT-23Q–HTT-74Q system in comparison to the oligomerization in the HTT-23Q–HTT-23Q system where it is absent (see Fig. 1g). Hence, we analyze important H-bond interactions between 23Q and 74Q subunits and perform ΔΔ*G* calculations on the most relevant cases. Residue Q175 located in the polyQ region of HTT-74Q has strong H-bond interactions with R17 of HTT-23Q, specifically with residues L64 and P65. Further, residues Q37 and Q38, located in the polyQ of HTT-23Q, have H-bond interactions with residues in the P11 region of HTT-74Q, P191 and P192 respectively. In addition Q38 displays H-bond interaction with L194 residue in R17 region of HTT-74Q.

**Figure 3:**
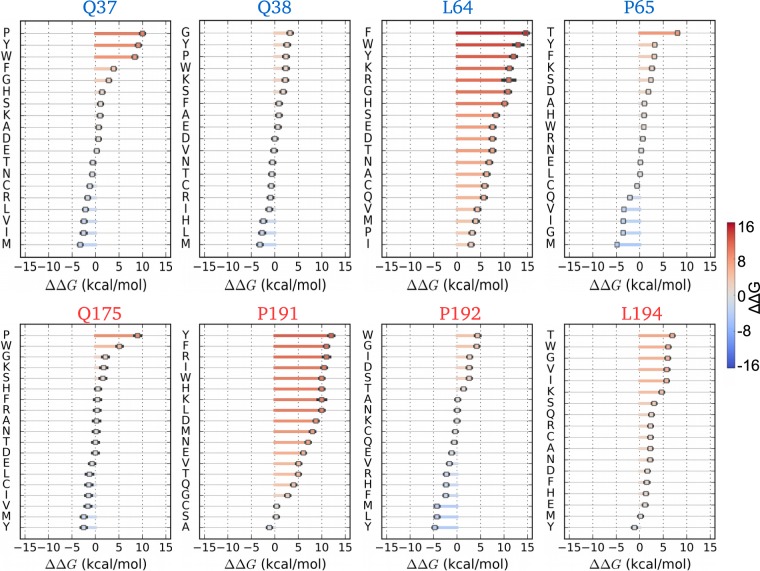
Selected amino acid induced mutations can destabilize the HTT-23Q–HTT-74Q oligomers. Destabilizing (stabilizing) mutations are marked with red (blue) dots. Residue Q175 located in the polyQ region of HTT74Q has strong H-bond interactions with R17 of HTT23Q, specifically with residues L64 and P65. Further, residues Q37 and Q38, located in the polyQ of HTT-23Q, have H-bond interactions with residues in the P11 region of HTT-74Q, P191 and P192 respectively. In addition Q38 displays H-bond interaction with L194 residue in R17 region of HTT-74Q (aggregate shown in Fig. 1i). Blue labels refer to mutation of the amino acids belonging to HTT-23Q while red ones to HTT-74Q. The green axis label correspond to residues located in polyQ region of HTT-23Q, while the magenta labels to R12 region of the mutant HTT-74Q (oligomer shown in Fig. 1i). Error bars are ±standard deviation.

As shown in Fig. 3, substitution of the W residue at all the selected positions mostly induces destabilization on the oligomer structure due to its bulky and aromatic side chain. Furthermore, calculations on Q37 (Fig. 3a) and Q38 (Fig. 3b), residues show a similar trend in stabilization/destabilization activity on the quaternary structure of the aggregate. In particular, the substitution of residues such as M, I and Y induces significant stability in the HTT-23Q–HTT-74Q aggregate, while the substitution of G at all chosen positions in the polyQ regions has a significant destabilization effect. Interestingly, mutation of Q37 to P37 shows a remarkable disruption effect on the assembly of HTT-23Q–HTT-74Q oligomer structures. Furthermore, Eris calculations on residue Q175 positioned in the polyQ of HTT-74Q display similar stabilization/destabilization effects with all amino acids, to Q37 especially when substituting a P residue. The insertion of either G or W residue in place of P192 and L194 has deleterious effects on the quaternary structure of the HTT-23Q–HTT-74Q oligomer. Furthermore we observe that almost all mutations at residue position P191 and L64 have destabilized the structure of HTT-23Q–HTT-74Q, which suggests the significance of this two residues at the oligomer’s interface. Finally, we perform a similar analysis for the HTT-74Q–HTT-74Q binary system and for the HTT-23Q–HTT-23Q–HTT-74Q ternary systems. The results are reported in Figs S4 and S5, respectively.

### FLIM-FRET experiments indicate heterogeneous HTT aggregation in cells

**Figure 4:**
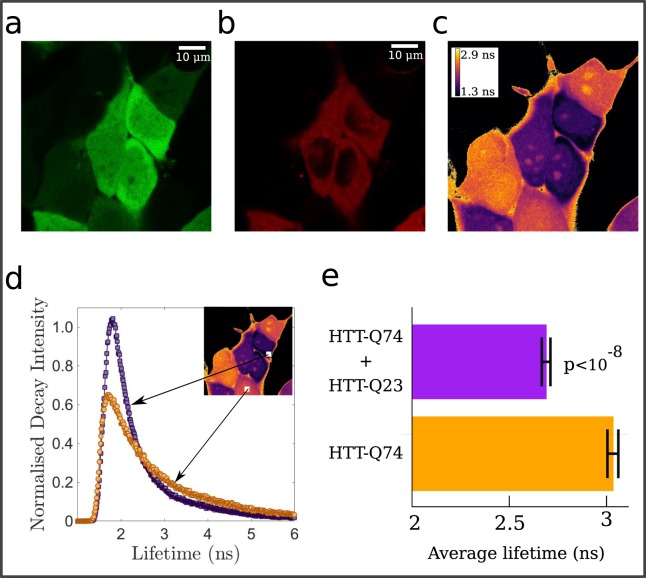
FLIM-FRET analysis shows heterogeneous aggregation of mutant and wild type HTT. Exemple of EGFP-HTT-74Q (**a**) and RFP-HTT-20Q (**b**) signal in HeLa cells. (**c**) Average EGFP lifetime obtained fitting single-pixel distribution with four exponents. Decay time in pixel with less than 150 counts was set to zero. (**d**) The decay signal distributions from two regions (30 × 30 pixels) show different exponential tails, in agreement with average lifetime estimates. (**e**) Average lifetimes calculated from lifetime distribution over regions of 30 × 30 pixels in 11 cells positive to EGFP-HTT-74Q and 14 cells positive to both EGFP-HTT-74Q and RFP-HTT-20Q transfection.

To confirm that aggregation between mutant and wild type HTT proteins occurs in cells, we cotransfect HeLa cells with PRK-RFP-exon1 HTT with 20Q, as a proxy for wild type HTT, and with pEGFP-exon 1 HTT with 74Q, representing mutated HTT. In these conditions, HeLa cells express both wild type and aberrant proteins linked to two distinct fluorochromes, as shown in Fig. 4a,b. Since fluorescence colocalization does not guarantee that the two proteins are in close proximity, we perform a FLIM-FRET analysis^28^. With this technique it is possible to estimate the fluorescence decay time of a donor molecule following a pulse of excitation time. When the donor and the acceptor are closer than 10 nm, the signal results in a decrease of the fluorescence lifetime of the donor molecule.

Figure 4c shows a clear decrease in the average lifetime in the regions where the two types of HTT proteins are co-localized (Fig. 4c) and a corresponding change in the lifetime distribution (Fig. 4d) and average lifetime (Fig. 4e). This result implies that two HTT proteins with polyQ of different lengths are found in very close proximity and are thus likely to form an oligomer or an aggregate.

## Discussion

Huntington’s disease is caused by an autosomal dominant mutation in the HTT gene leading to progressive neurodegeneration associated with protein aggregation in the nuclei of neuronal cells. Aggregation happens usually when the HTT protein contains an expanded polyQ region with more than 36 repeats. While the precise role of these aggregates is still debated in the scientific literature, their accumulation is a prerequisite for neurotoxicity, as recently reviewed^29^. Hence, detailed understanding on molecular mechanisms underlying HTT aggregation is necessary to identify possible peptides that may interfere with the process^5^. Most of the accumulated knowledge in this area refers to homogeneous aggregation of HTT with extended polyQ regions, while much less is known about heterogeneous aggregation of mutant and wild type forms of the protein. The possibility of heterogeneous aggregation was revealed more than a decade ago by experiments *in vitro*^18^ but received a renewed attention only in recent years thanks to recent experiments showing transcellular propagation of mutated HTT proteins in the *Drosophila*^9,10^ and mouse brain^8^.

In this paper, we explored molecular mechanism behind the unconventional HTT aggregation through MD simulations and compared heterogeneous and homogeneous oligomerization of HTT proteins. Our computational analyses complemented by experimental studies using FLIM-FRET have efficiently unraveled the mechanism of heterogeneous oligomerization. We deciphered how mutated HTT proteins could catalyze the aggregation process by generating strong HTT oligomers, comprising wild-type HTT proteins as well. This catalytic activity of HTT mutants was efficiently captured by our *in silico* studies where the interaction of two HTT wild-type proteins with a single HTT mutants leads to the formation of oligomers.

To corroborate our numerical results experimentally, we performed the FLIM-FRET analysis on cells co-transfected with wild type and mutant HTT. Previous experimental evidence of heterogeneous HTT aggregation and oligomerization was based on polymerization assays *in vitro*^18^ and fluorescence co-localization in cell lines^18^ and in animal models^9^. The FLIM-FRET technique allows to obtain a more convincing proof of intracellular aggregation since it is able to detect close proximity with greater sensitivity and precision than co-localization assays.

Having identified the structural mechanisms for HTT oligomerization, we identified hot spot residues that trigger the aggregation process through ΔΔ*G* calculations^27^. Using this strategy, we were able to identify molecular hot spots which could become possible targets to induce disaggregation and contrast the disease. According to our analysis the residues contributing to the heterogeneous oligomerization typically differ from those determining conventional oligomerization. This issue is important for the definition of molecular targets to inhibit aggregation, particularly in view of a possible prion-like propagation of Huntington’s disease^12,13^ that is emerging as a common feature in many neurodegenerative disorders, including Alzheimner’s and Parkinson’s diseases^30^.

## Materials and Methods

### Simulations protocol

We use Discrete Molecular Dynamics (DMD) where the all-atom protein model interacts through the implicit solvent force-field Medusa which includes the van der Waals interaction, hydrogen bonds, electrostatic, dihedral and angular interactions and those due to the effective solvent. We perform replica exchange simulations to efficiently sample the energy landscape of the proteins^31^. For each simulated system, we carry out simulations using 20 replicas of the system with temperature *T* in the range of 0.5–0.72 simulation units (this range has been previously investigated in^16^). We started each simulation from completely stretched conformations, discarding the first part of the trajectories, that were not equilibrated due to the initial high energy. For the analysis, we only considered equilibrated trajectories (see Fig. S1). At a regular time interval of 1000 time units (approximately 50 ps), we exchange the temperatures of two replicas, also in line with previous work^16^. The duration of the simulation of each replica is about 4 × 10^6^ time units (200 ns). For the analysis, we only consider equilibrated trajectories. A cubic box with periodic boundary condition has been employed for each simulated system. The analysis is performed using the GROMACS utility^32^; the secondary structures are computed using STRIDE^33^ and visualized with VMD^34^. Error bars on the estimated *β*-structure probabilities are obtained assuming binomial statistics in the normal approximation yielding ${\rm{\Delta }}p=z{(p\mathrm{(1}-p)/N)}^{\mathrm{1/2}}$, where *p* is the estimated probability, *z* = 1.96 for a 95% confidence level and *N* is the number of sampled configurations. In our simulations, *N* = 4000 so that the upper bound on the error bar is ${\rm{\Delta }}{p}^{max}=0.015$.

### Aggregation maps: average beta structures between two proteins

To perform *β*-cross interaction analysis we consider pairwise systems, searching for beta structures and in the second one. If their distance is less than 0.4 nm we measure the number of contacts between any pair of atoms from the respective group. We then average over the number of trajectory frames.

### Cluster analysis: most probable structures

To determine the most probable structure of the target system, we perform cluster analysis using the single linkage method^26^ using the single linkage method implemented in GROMACS^32^. We add a structure to a cluster when its distance to any element of the cluster is less than a cutoff, chosen to be 4 *Å*.

### Estimation of aggregate stability

Important interactions between different binding partners are found using the Eris molecular design suite^27^. Briefly, Eris substitutes all possible amino acids at chosen position sequentially and re-packs the side chains of the residues surrounding the mutated site each time using a Monte Carlo simulated annealing procedure. Utilizing Medusa force field, Eris calculates the change of protein stability induced by the mutations with respect to the wild type protein in terms of ${\rm{\Delta }}{\rm{\Delta }}G={\rm{\Delta }}{G}_{mutant}-{\rm{\Delta }}{G}_{WT}$. Thus, positive and negative ΔΔ*G* values signify destabilizing and stabilizing mutations respectively. It is worth to mention here that the substitutions involving proline residues have resulted in heuristic values due to its cyclic structure and conformational rigidity. Hence, proline substitutions are omitted from most of the systems in the present study. Calculations are averaged over 250 iterations.

### Cell culture and transfection

HeLa cells were maintained in DMEM (Euroclone, cod. ECM0060L) medium with 10% fetal bovine serum (Euroclone, ECS0180L), 100 U/ml penicillin, 100 mg/ml streptomycin sulphate (Euroclone, cod. ECB3001D) and 2 mM L-Glutamine (Euroclone, cod. ECB3000D-20) at 37 °C in an atmosphere of 5% CO2 and 95% humidity. Cells seeded at an 70% confluent onto 6-well plates were transiently transfected with PRK-RFP-exon1 HTT with 20Q (kindly provided from S. H. Li Laboratory, Emory University)^35^ or pEGFP-exon 1 HTT with 74Q (Addgene, 40262) or cotrasfected with PRK-RFP-exon1 HTT with 20Q and pEGFP-exon 1 HTT with 74Q plasmids using Xfect transfection reagent (Clontech, cod 631317). After 48 h from transfection cells were used for live FLIM-FRET analysis.

### Imaging and FLIM-FRET analysis

The FLIM-FRET technique allows to estimate the fluorescence decay time of a donor molecule following a pulse of excitation light^28^. Spectrally distinct fluorophores were chosen in such a way that the emission spectrum of the donor molecule (EGFP) overlaps the excitation spectrum of the acceptor molecule (RFP). When closer less than approximately 10 nm, the excited donor can transfer its energy to the acceptor, resulting in a decrease of the fluorescence lifetime of the donor molecule. Images were acquired with Leica TCS SP8 microscope and PicoQuant modules for Time-Correlated Single Photon Counting (TCSPC) in time-tagged time-resolved (TTTR) mode using 60x objective. The EGFP donor fluorophore was excited at 491 nm at a laser pulse frequency of 19.5 MHz. Average lifetime was calculated using FlimFit (v.5.1.1)^36^, Fiji-ImageJ (v.1.52e)^37,38^ PTUreader plugin (v.0.06). Biexponential function was used to fit pure EGFP lifetime, while for EGFP-RFP interacting fluorophores four exponential were used. Lifetimes were calculated on each pixel using a count threshold of 150. The instrument response function function was estimated directly from acquired images. Statistical analysis of the average lifetime was performed using 11 cells positive to EGFP-HTT-74Q and 14 cells positive to both EGFP-HTT-74Q and RFP-HTT-20Q transfection, obtained from two independent experiments. Statistical significance was established using the unpaired t-test.

## Supplementary information

Supplementary Information

## Data Availability

All data generated or analysed during this study are included in this published article (and its Supplementary Information files).

## References

[1] Davies SW (1997). Formation of neuronal intranuclear inclusions underlies the neurological dysfunction in mice transgenic for the hd mutation. Cell.

[2] Walker FO (2007). Huntington’s disease. Lancet.

[3] Rubinsztein DC (1996). Phenotypic characterization of individuals with 30–40 cag repeats in the huntington disease (hd) gene reveals hd cases with 36 repeats and apparently normal elderly individuals with 36–39 repeats. American journal of human genetics.

[4] Kar K, Jayaraman M, Sahoo B, Kodali R, Wetzel R (2011). Critical nucleus size for disease-related polyglutamine aggregation is repeat-length dependent. Nat Struct Mol Biol.

[5] Chen M, Wolynes PG (2017). Aggregation landscapes of huntingtin exon 1 protein fragments and the critical repeat length for the onset of huntington’s disease. Proceedings of the National Academy of Sciences.

[6] Mangiarini L (1996). Exon 1 of the hd gene with an expanded cag repeat is sufficient to cause a progressive neurological phenotype in transgenic mice. Cell.

[7] DiFiglia M (1995). Huntingtin is a cytoplasmic protein associated with vesicles in human and rat brain neurons. Neuron.

[8] Pecho-Vrieseling E (2014). Transneuronal propagation of mutant huntingtin contributes to non-cell autonomous pathology in neurons. Nat Neurosci.

[9] Pearce MMP, Spartz EJ, Hong W, Luo L, Kopito RR (2015). Prion-like transmission of neuronal huntingtin aggregates to phagocytic glia in the drosophila brain. Nat Commun.

[10] Babcock DT, Ganetzky B (2015). Transcellular spreading of huntingtin aggregates in the drosophila brain. Proc Natl Acad Sci USA.

[11] Jansen AHP, Batenburg KL, Pecho-Vrieseling E, Reits EA (2017). Visualization of prion-like transfer in huntington’s disease models. Biochim Biophys Acta Mol Basis Dis.

[12] Cicchetti F (2014). Mutant huntingtin is present in neuronal grafts in huntington disease patients. Ann Neurol.

[13] Jeon I (2016). Human-to-mouse prion-like propagation of mutant huntingtin protein. Acta Neuropathol.

[14] Chen S, Ferrone FA, Wetzel R (2002). Huntington’s disease age-of-onset linked to polyglutamine aggregation nucleation. Proc Natl Acad Sci USA.

[15] Bhattacharyya AM, Thakur AK, Wetzel R (2005). polyglutamine aggregation nucleation: thermodynamics of a highly unfavorable protein folding reaction. Proc Natl Acad Sci USA.

[16] Lakhani VV, Ding F, Dokholyan NV (2010). Polyglutamine induced misfolding of huntingtin exon1 is modulated by the flanking sequences. PLoS Comput Biol.

[17] Chen M, Tsai M, Zheng W, Wolynes PG (2016). The aggregation free energy landscapes of polyglutamine repeats. J Am Chem Soc.

[18] Busch A (2003). Mutant huntingtin promotes the fibrillogenesis of wild-type huntingtin: a potential mechanism for loss of huntingtin function in huntington’s disease. J Biol Chem.

[19] Isas JM, Langen A, Isas MC, Pandey NK, Siemer AB (2017). Formation and structure of wild type huntingtin exon-1 fibrils. Biochemistry.

[20] Długosz M, Trylska J (2011). Secondary structures of native and pathogenic huntingtin n-terminal fragments. The Journal of Physical Chemistry B.

[21] Bulone D, Masino L, Thomas DJ, San Biagio PL, Pastore A (2006). The interplay between polyq and protein context delays aggregation by forming a reservoir of protofibrils. PLoS One.

[22] Piana S, Lindorff-Larsen K, Shaw DE (2012). Protein folding kinetics and thermodynamics from atomistic simulation. Proceedings of the National Academy of Sciences.

[23] Ding F, Tsao D, Nie H, Dokholyan NV (2008). Ab initio folding of proteins with all-atom discrete molecular dynamics. Structure.

[24] Proctor EA, Ding F, Dokholyan NV (2011). Discrete molecular dynamics. Wiley Interdisciplinary Reviews: Computational Molecular Science.

[25] Dokholyan NV (2006). Studies of folding and misfolding using simplified models. Current opinion in structural biology.

[26] Gan, G., Ma, C. & Wu, J. *Data clustering: theory, algorithms, and applications*, vol. 20 (Siam, 2007).

[27] Yin S, Ding F, Dokholyan NV (2007). Eris: an automated estimator of protein stability. Nature Methods.

[28] De Los Santos C, Chang C-W, Mycek M-A, Cardullo RA (2015). Frap, flim, and fret: detection and analysis of cellular dynamics on a molecular scale using fluorescence microscopy. Molecular reproduction and development.

[29] Zhao T, Hong Y, Li X-J, Li S-H (2016). Subcellular clearance and accumulation of huntington disease protein: A mini-review. Front Mol Neurosci.

[30] Stopschinski BE, Diamond MI (2017). The prion model for progression and diversity of neurodegenerative diseases. Lancet Neurol.

[31] Sugita Y, Okamoto Y (1999). Replica-exchange molecular dynamics method for protein folding. Chemical physics letters.

[32] Hess B, Kutzner C, Van Der Spoel D, Lindahl E (2008). Gromacs 4: algorithms for highly efficient, load-balanced, and scalable molecular simulation. Journal of chemical theory and computation.

[33] Frishman D, Argos P (1995). Knowledge-based protein secondary structure assignment. Proteins: Structure, Function, and Bioinformatics.

[34] Humphrey W, Dalke A, Schulten K (1996). Vmd: visual molecular dynamics. Journal of molecular graphics.

[35] Li X (2010). Inhibiting the ubiquitin–proteasome system leads to preferential accumulation of toxic n-terminal mutant huntingtin fragments. Human Molecular Genetics.

[36] Warren, S. *et al*. Rapid global fitting of large fluorescence lifetime imaging microscopy datasets. *PLoS One* **8** https://www.scopus.com/inward/record.uri?eid=2-s2.0-84881105876&doi=10.1371%2fjournal.pone.0070687&partnerID=40&md5=677816dede4fc243d224bdab7fc4e415 (2013). 10.1371/journal.pone.0070687 PMC3734241 23940626

[37] Schneider, C., Rasband, W. & Eliceiri, K. Nih image to imagej: 25 years of image analysis. *Nature Methods* **9**, 671–675, https://www.scopus.com/inward/record.uri?eid=2-s2.0-84863205849&doi=10.1038%2fnmeth.2089&partnerID=40&md5=27f8386cad59f4df66133961b6164bae (2012). 10.1038/nmeth.2089 PMC5554542 22930834

[38] Schindelin, J. *et al*. Fiji: An open-source platform for biological-image analysis. *Nature Methods* **9**, 676–682, https://www.scopus.com/inward/record.uri?eid=2-s2.0-84862520770&doi=10.1038%2fnmeth.2019&partnerID=40&md5=4f86c2ea85d9ca9f1539ffbfc2b7f403 (2012). 10.1038/nmeth.2019 PMC3855844 22743772

