# Supplementary material for: Molecular mechanisms of heterogeneous oligomerization of huntingtin proteins
Source: Sci Rep. 2019 May 20;9:7615. doi: 10.1038/s41598-019-44151-0 (PMC6527588; doi:10.1038/s41598-019-44151-0)
Supplement: Supplementary file 1 — Supplementary Information [file 41598_2019_44151_MOESM1_ESM.pdf]

Supplementary Information for ”Molecular mechanisms of heterogeneous oligomerization of huntingtin proteins”

Silvia Bonfanti[1], Maria Chiara Lionetti[2], Maria Rita Fumagalli[2], Venkat R. Chirasani[3], Guido Tiana[1], Nikolay V. Dokholyan[3], Stefano Zapperi[1,4], and Caterina A. M. La Porta[2,*]

[1]Center for Complexity and Biosystems, Department of Physics, University of Milan, Via Celoria 26, 20133 Milano, Italy
[2]Center for Complexity and Biosystems, Department of Environmental Science and Policy, University of Milano, via Celoria 26, 20133 Milano, Italy
[3]Departments of Pharmacology and Biochemistry & Molecular Biology, Penn State College of Medicine, PA 17033, USA
[4]CNR - Consiglio Nazionale delle Ricerche, Istituto di Chimica della Materia Condensata e di Tecnologie per l'Energia, Via R. Cozzi 53, 20125 Milano, Italy
[*]Corresponding author: caterina.laporta@unimi.it

ABSTRACT

There is still no successful strategy to treat Huntington's disease, an inherited autosomal disorder associated with the aggregation of mutated forms of the huntingtin protein containing polyglutamine tracts with more than 36 repeats. Recent experimental evidence is challenging the conventional view of the disease by revealing transcellular transfer of mutated huntingtin proteins which are able to seed oligomers involving wild type forms of the protein. Here we decipher the molecular mechanism of this unconventional heterogeneous oligomerization by performing discrete molecular dynamics simulations. We identify the most probable oligomer conformations and the molecular regions that can be targeted to destabilize them. Our computational findings are complemented experimentally by fluorescence-lifetime imaging microscopy/fluorescence resonance energy transfer (FLIM-FRET) of cells co-transfected with huntingtin proteins containing short and large polyglutamine tracts. Our work clarifies the structural features responsible for heterogeneous huntingtin aggregation with possible implications to contrast the prion-like spreading of Huntington's disease.

Supplementary Text

Amino acid sequences

We report below the amino acid sequences for the two forms of huntingtin first exon studied here:

HTT-23Q:
MATLEKLMKAFESLKSF
QQQQQQQQQQQQQQQQQ
QQQQQQPPPPPPPPPPP
QLPQPPPQAQPLLPQPQP
PPPPPPPPPGPAVAEEPLHRP

HTT-74Q:
MATLEKLMKAFESLKSFQQQQQQQQQ
QQQQQQQQQQQQQQQQQQQQQQQ
QQQQQQQQQQQQQQQQQQQQQQQQQ
QQQQQQQQQQQQQQQQQ
PPPPPPPPPPPQLPQPPPQ
AQPLLPQPQPPPPPPPPPPG
PAVAEEPLHRP

Equilibration of discrete molecular dynamics simulations

Time evolution of the energies for the different temperatures from equilibrated DMD simulations (left panels of Figure S1). Equilibration is ensured by the absence of significant energy drift. The distributions of the energies obtained for the replicas at the different temperatures (right panels of Fig. S1) show overlap among neighboring temperatures which is needed for the replica exchange method. The temperatures for each simulated system are indicated in the legend.

Supplementary Figures

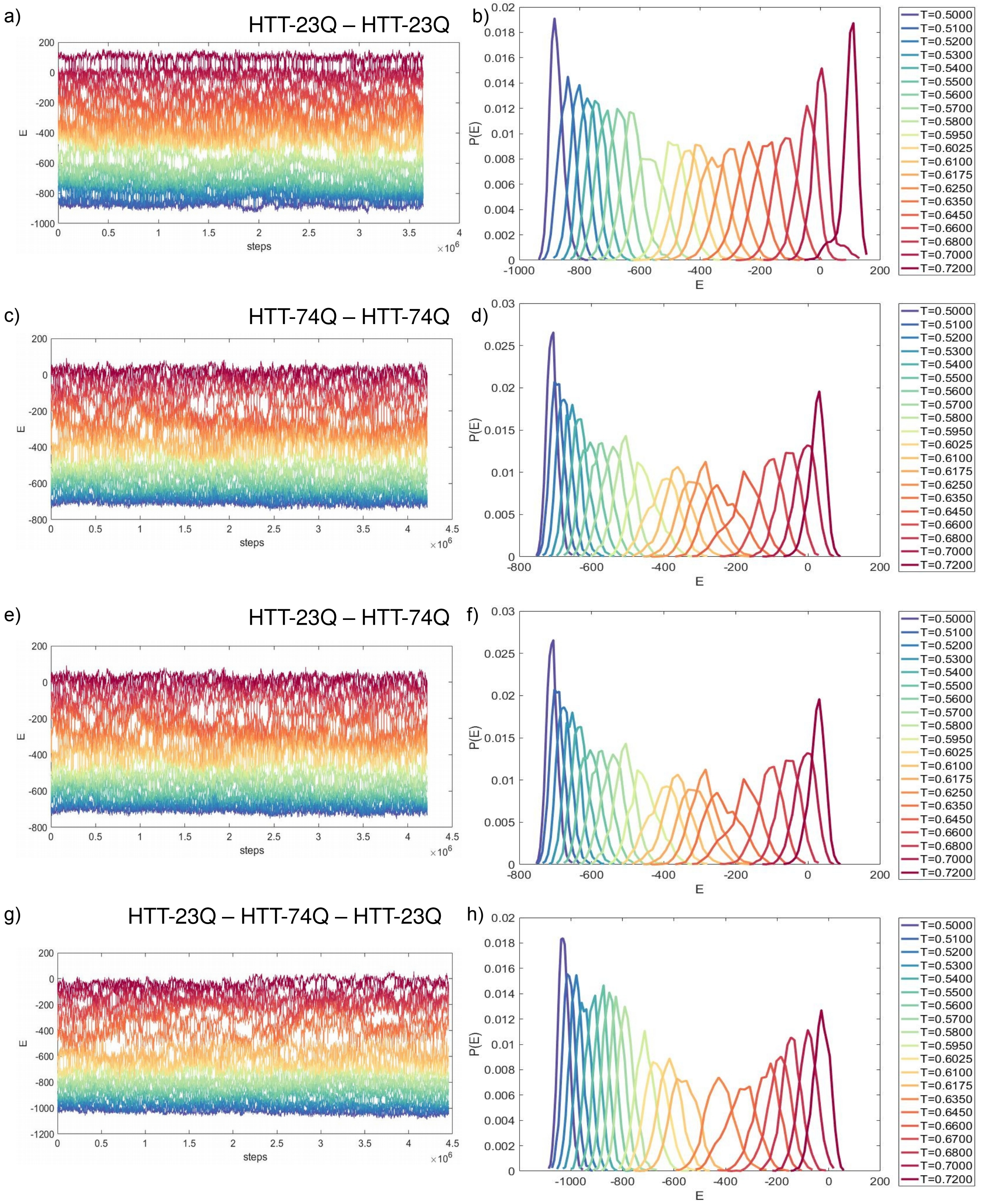

Figure S1. Equilibration of DMD simulations. a,c,e,g) Time evolution of the energies for the different temperatures from equilibrated DMD simulations for the four systems simulated in this work: a) HTT-23Q−HTT-23Q, b) HTT-74Q−HTT-74Q, c) HTT-23Q−HTT-74Q d) HTT-23Q−HTT-23Q−HTT-74Q. b,d,e,f) Distribution of the energy obtained for the replicas at the different temperatures indicated in the legend.

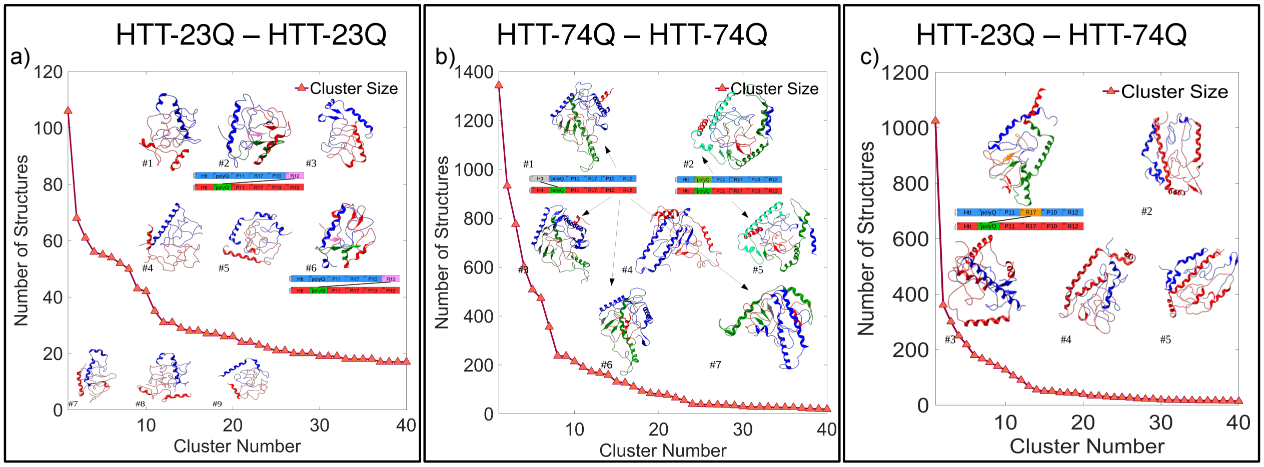

Figure S2. **Cluster Analysis: number of structures found for each cluster for the three binary systems.** a) HTT-23Q−HTT-23Q, b) HTT-74Q−HTT-74Q, c) HTT-23Q−HTT-74Q. The central conformational structures for the first most probable clusters are shown.

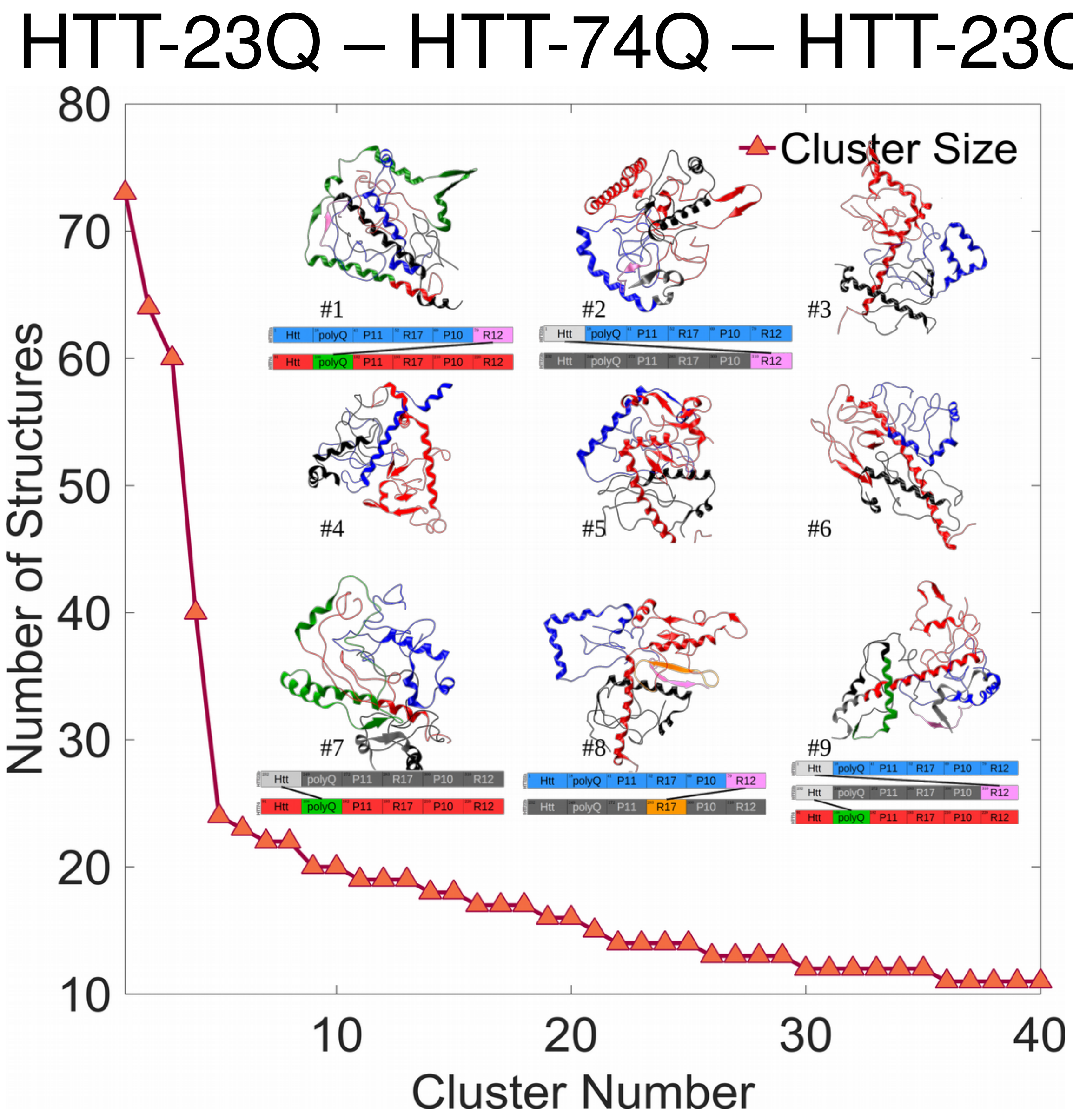

Figure S3. **Cluster Analysis: number of structures found for each cluster for the ternary system: HTT-23Q−HTT-23Q−HTT-74Q.** The central conformational structures for the first most probable clusters are shown.

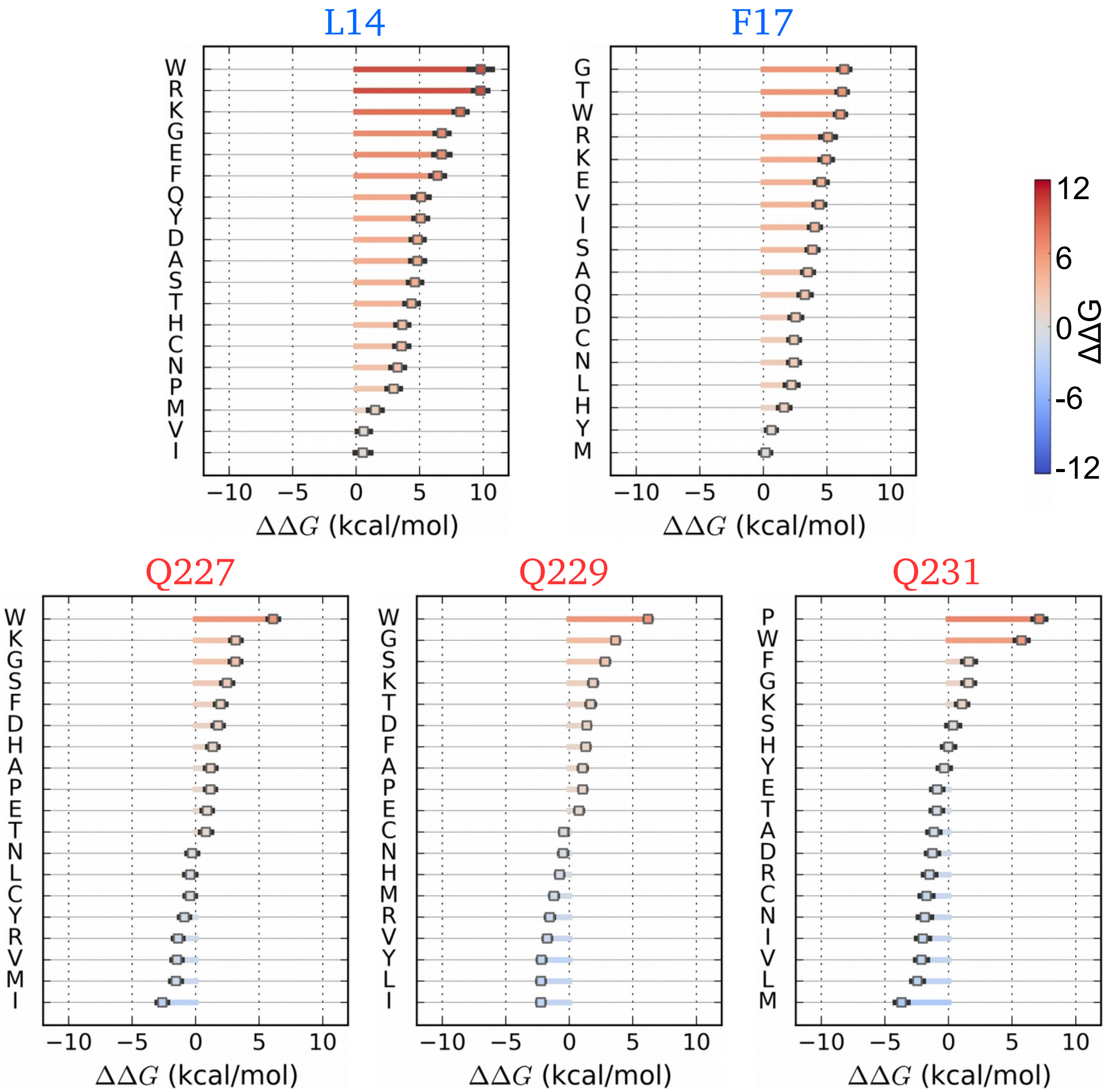

Figure S4. Selected amino acid induced mutations can destabilize the HTT-74Q−HTT-74Q oligomer. Amino acids induced mutations that can destabilize (stabilize) the oligomer HTT-74Q−HTT-74Q are marked with red (blue) dots. Residue L14 in Htt region of one protein has two H-bond interactions with the polyQ region residues Q227 and Q229 of the other protein. Furthermore, F17 in Htt domain is also involved in two H-bond interactions with the polyQ region residues Q229 and Q231 (oligomer structure shown in Figure 1h). Blue labels refer to mutation of the amino acids belonging to first HTT-74Q while red ones to the other HTT-74Q. Error bars are ± standard deviations.

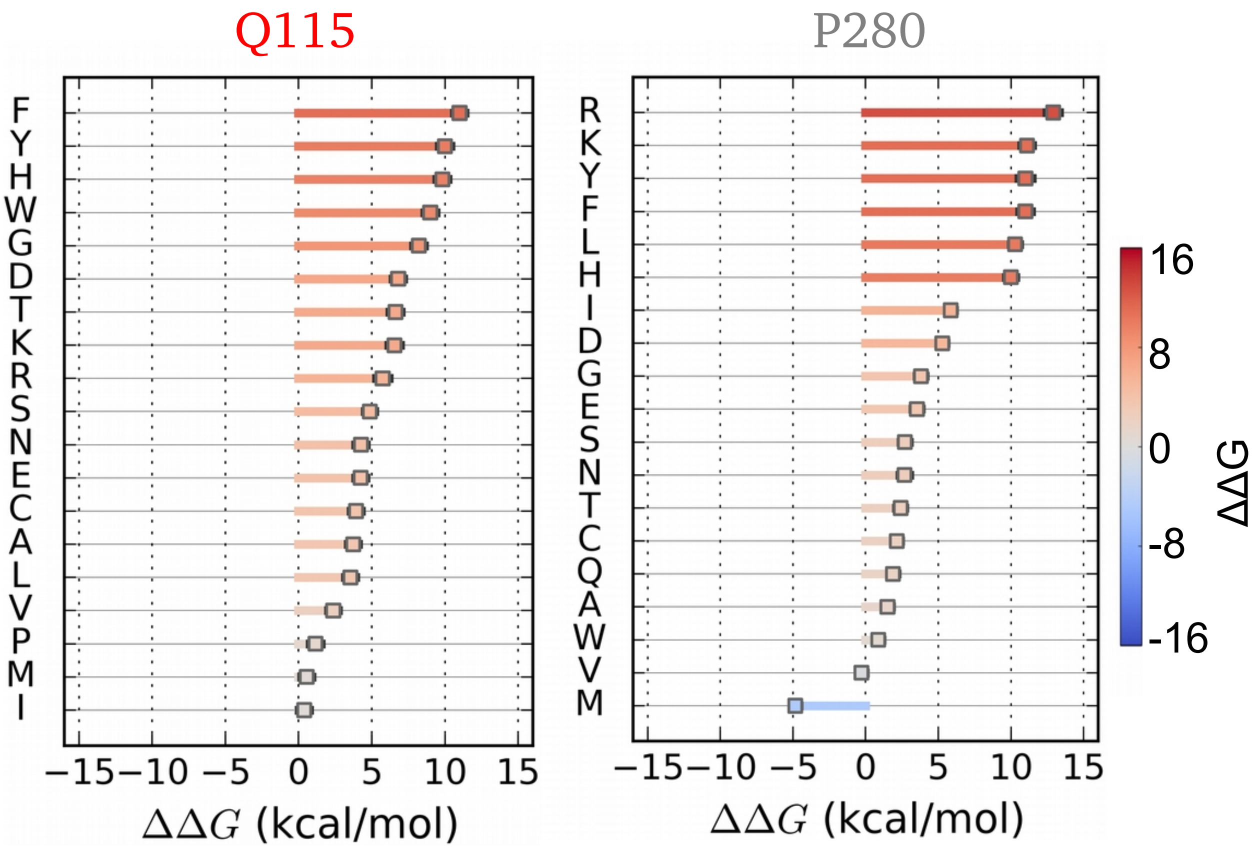

Figure S5. Selected amino acid induced mutations can destabilize the HTT-23Q−HTT-23Q−HTT-74Q trimer. The main effect is seen in the residues Q115 and P280. Error bars are ± standard deviations.
